# Development of a Target-to-Sensor Mode Multispectral Imaging Device for High-Throughput and High-Precision Touch-Based Leaf-Scale Soybean Phenotyping

**DOI:** 10.3390/s23073756

**Published:** 2023-04-05

**Authors:** Xuan Li, Ziling Chen, Xing Wei, Tianzhang Zhao, Jian Jin

**Affiliations:** Department of Agricultural and Biological Engineering, Purdue University, West Lafayette, IN 47907, USA

**Keywords:** image-based plant phenotyping, precision agriculture, automated system, proximal sensor, active sensing, airflow

## Abstract

**Highlights:**

**What are the main findings?**

- Innovatively designed sensor that uses airflow to adaptively reposition and flatten soybean leaves for optimized imaging results.
- The developed device can identify the effect of nitrogen treatment under both controlled environments and field conditions.

**What is the implication of the main finding?**

- The throughput and resolution of obtaining a multispectral soybean image has been elevated compared to current proximal whole leaf imagers.
- Proximal sensing has the potential to outperform remote sensing because of the higher signal-over-noise ratio.

**Abstract:**

Image-based spectroscopy phenotyping is a rapidly growing field that investigates how genotype, environment and management interact using remote or proximal sensing systems to capture images of a plant under multiple wavelengths of light. While remote sensing techniques have proven effective in crop phenotyping, they can be subject to various noise sources, such as varying lighting conditions and plant physiological status, including leaf orientation. Moreover, current proximal leaf-scale imaging devices require the sensors to accommodate the state of the samples during imaging which induced extra time and labor cost. Therefore, this study developed a proximal multispectral imaging device that can actively attract the leaf to the sensing area (target-to-sensor mode) for high-precision and high-throughput leaf-scale phenotyping. To increase the throughput and to optimize imaging results, this device innovatively uses active airflow to reposition and flatten the soybean leaf. This novel mechanism redefines the traditional sensor-to-target mode and has relieved the device operator from the labor of capturing and holding the leaf, resulting in a five-fold increase in imaging speed compared to conventional proximal whole leaf imaging device. Besides, this device uses artificial lights to create stable and consistent lighting conditions to further improve the quality of the images. Furthermore, the touch-based imaging device takes full advantage of proximal sensing by providing ultra-high spatial resolution and quality of each pixel by blocking the noises induced by ambient lighting variances. The images captured by this device have been tested in the field and proven effective. Specifically, it has successfully identified nitrogen deficiency treatment at an earlier stage than a typical remote sensing system. The *p*-value of the data collected by the device (*p =* 0.008) is significantly lower than that of a remote sensing system (*p =* 0.239).

## 1. Introduction

### 1.1. Background of Image-Based Plant Phenotyping

Plant phenotyping is a critical tool for understanding the complex interplay between genomics, environment and management (G × E × M). In particular, multispectral and hyperspectral imaging technologies offer rich spectral and spatial data, making them valuable for studying plant traits [1]. Nowadays, advanced imaging systems and devices have been deployed to collect multi- and hyperspectral phenomics data [2] and the imaging systems can be categorized as remote sensing and proximal sensing based on the imaging distance [3].

Remote sensing technologies offer powerful tools for studying plant traits, with unmanned aerial vehicles (UAVs) being one widely used option. UAVs can be flown between 5 and 200 m above the field, allowing for the capture of crop spectra [4]. Studies using UAVs have demonstrated their ability to analyze a range of species and traits [5,6,7,8,9,10,11]. Alternatively, ground-based vehicles and gantry systems offer higher spatial resolution due to their shorter imaging distance compared to UAVs [12,13,14,15]. These studies prove the effectiveness of remote sensing as a method for multispectral imaging; however, these methods are not promising for capturing high-resolution and high-signal-over-noise multispectral images. Firstly, the UAV systems use sunlight as the source of full-spectrum light that has varying intensity at different time and different location [16]; thus, such characteristics compromise the reflectance spectrum of the canopies and result in non-accurate and non-consistent multispectral images. Moreover, the morphological structure of canopies causes shades, bright spots and different imaging angle for individual leaf. These factors impose noises that strongly impact the imaging results. Lastly, the UAVs can only produce plot-level or canopy-level multispectral image, which does not release the potential of precise phenotyping as abiotic stress is not uniformly distributed among the plants [17,18] and biotic stress will also first appear on leaves [19,20]. Therefore, it is necessary to capture more detailed images and more precise spectrum of the plants through proximal sensing. 

Proximal sensors, including soil plant analysis development (SPAD) meters, are commonly used to monitor plant growth and health condition. Multiple studies have shown that SPAD readings are correlated with various crop traits [21,22,23]. However, since the SPAD meter only measures a small area of the leaf, different locations on the same leaf can yield different results [17,24] Additionally, studies have suggested that color distribution over the entire leaf is a better indicator of stress [25,26].

Therefore, imaging devices that capture the entire leaf are crucial and valuable for advancing plant science research. Such devices can provide researchers with a more comprehensive view of the plant and help to reveal insights into the complex interactions between crops and their environment.

### 1.2. Related Work

Recently, handheld multispectral and hyperspectral whole leaf imaging devices have been developed and proved useful for determining nutrient stress. Zhang et al. [18] developed a multispectral corn leaf imager for precise phenotyping. The spectrum from the multispectral imager showed a strong correlation with the SPAD meter readings. Also, for corn phenotyping, Wang et al. [27] developed a handheld hyperspectral imaging device. The device is designed to clamp the corn leaf between a full-spectrum light and the imaging system. The mean spectrum captured by the device successfully separated genotype and nitrogen treatment. Similarly, Wang, Duan et al. [28] developed a multispectral imager, which separated two genotypes of soybean from different stresses using the images captured by the device, and the time for taking a multispectral image is 20 s. 

However, all existing leaf-level multispectral imaging devices require sophisticated operation. Specifically, the users need to approach the sensor to the leaf, then flatten the leaf and clamp it between the light source and the camera to capture images. This operation is time-consuming and laborious, regardless of whether it is in the field or in the controlled environment. This traditional sensor-to-target imaging mode needs to be redesigned to reach higher throughput. Therefore, it is necessary to improve the fundamental mechanisms of these leaf-level imagers to relieve the labor and operation cost to increase the throughput while taking full advantage of proximal sensing. Furthermore, the device also needs to provide consistent and stable lighting conditions to remove the noises induced by the environment.

### 1.3. Scope of This Paper

With the considerations described previously, in this work, we present an innovated multispectral leaf-level imaging device for dicotyledon plant leaves that is fast and accurate. This device is designed to provide consistent lighting conditions by using LEDs while blocking out the ambient light. Such design is necessary to obtain informative images. Moreover, this device adopts a new mechanism that uses active air flow to manipulate the orientation of the leaf, keep the leaf flattened and hold the leaf in place against a leaf barrier made by ultra-fine nylon threads. This mechanism creates a new target-to-sensor mode, which relieves the users from clamping and flattening the leaf, thus, the throughput is increased by five times compared to other whole leaf imaging devices for dicotyledon plant. Furthermore, the device is compared with a remote sensing system to compare the capability of separating nitrogen treatments.

In this paper, Section 2 describes the hardware components of this device along with the workflow for operating this device. Section 3 explains the post-processing of the images captured by the device. The goal of this process is to improve image quality and consistency. After the device was ready, we created a task to validate its effectiveness. Section 4 includes the details and the results of the task involved distinguishing between nitrogen treatments using the images captured by the device. Section 5 discusses the new findings discovered in the study as well as specific challenges that need to be solved in the future. Lastly, the paper is concluded by Section 6.

## 2. Hardware Development

### 2.1. Hardware Configuration

**Figure 1:**
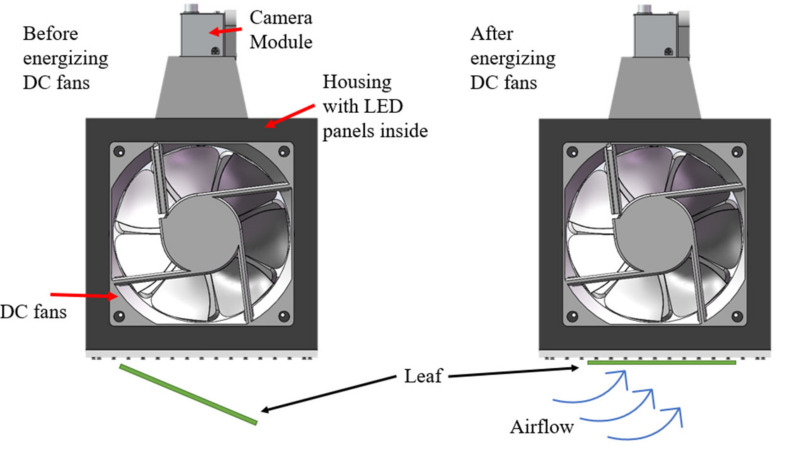
Device mechanism.

Figure 1 shows a schematic of the fundamental mechanism of the device. Assuming a leaf is initially tilting downside, by energizing the DC fans, the induced pressure difference will push the leaf towards the barrier and hold it against the barrier. This new mechanism will relieve the operators from capturing and holding the leaf while imaging and maintaining consistent imaging angle for all leaves.

**Figure 2:**
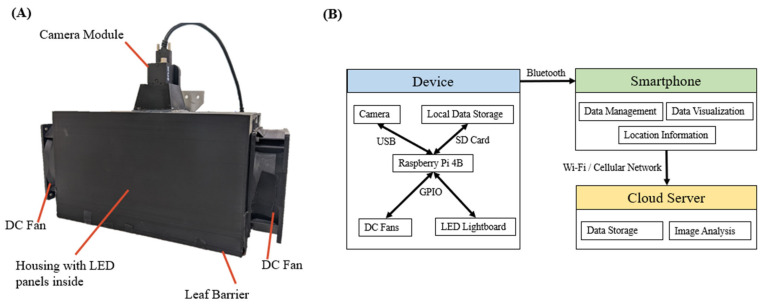
(**A**) Device Assembly. (**B**) Command and data flow between each component.

Figure 2A shows the assembled device with main components labeled in the figure. The dimension of the device is 346 mm × 150 mm × 225 mm (L × W × H), the imaging window is 150 mm × 120 mm (L × W). 

The DC fans used to create the airflow were manufactured by Mechatronics; each of the fans has a nominal 4500 rotation per minute (RPM) max under 12 V input.

The leaf barrier attached to the bottom of the imaging chamber consists of a grid of 0.2 mm thick nylon threads anchored to the frame. These fine monofilament nylon threads have low elasticity and high strength that allow them to hold the leaf flat and prevent the leaf from being sucked into the chamber. A black non-transparent felt fabric (150 mm × 225 mm) is attached to the leaf barrier, and it will sandwich the leaf after it is captured and hold against the nylon network to block the ambient light to reduce the noise.

This device images a single leaf to obtain the reflectance multispectral images of four wavelengths including blue, green, red and near infrared (NIR) bands. The wavelength of each band 460 nm (blue), 525 nm (green), 630 nm (red) and 850 nm (NIR). The image captured under visible light (red, green and blue) and NIR light is helpful to investigate the chlorophyll and nitrogen content. There are two light boards in the device, and each lightboard is covered with 2-mm thick white Teflon panels to diffuse the light for even distribution. Each light board houses eight LED strips (one strip per wavelength aligned sequentially as red, blue, green and NIR with one duplication) manufactured by Waveform Lightings.

A monochrome camera manufactured by Teledyne FLIR LLC is used to image the leaf. The camera has a resolution of 1920 pixels × 1200 pixels (H × V) and a pixel size of 5.86 um × 5.86 um (H × V). A low distortion (−1.0%) lens with fixed focal length manufactured by Kowa Optronics Co., Ltd. is attached to the camera as an object lens. 

The device is powered by a 12 V rechargeable portable battery. The power source is converted to 5 V to power the Raspberry Pi 4B, which serves as the microprocessor of the device. The LEDs and fans are powered by the same power source that the microprocessor uses, but the power is stabilized via a 12 V–12 V converter to maintain the brightness of the LEDs when the battery voltage decreases. A push button switch wired to the microprocessor is used to initiate the imaging sequence. The microprocessor, programmed using Python3, sends pulse-width modulation (PWM) signal to LED and fan drivers to turn on the LEDs and control the fans to operate at 80% of the max RPM. The microprocessor also powers the camera and controls the imaging timing through onboard USB 3.1 port. The images captured are saved as tagged image file format (.TIFF) in SD card mounted on Raspberry Pi 4B. The power consumption of the device is 30.7 W when imaging and 1.6 W while idling. 

The command and data flow are illustrated in Figure 2B. This device can record the location of each collected sample, which is helpful to create a field map for farmers to understand the field better. Moreover, this device uses a cloud server to permanently store the data and analysis the collected images, so there is almost no limitation on computing power and data size. This design allows users to easily manage the data and apply models freely.

### 2.2. Operation Flow for In-Vivo Imaging

**Figure 3:**
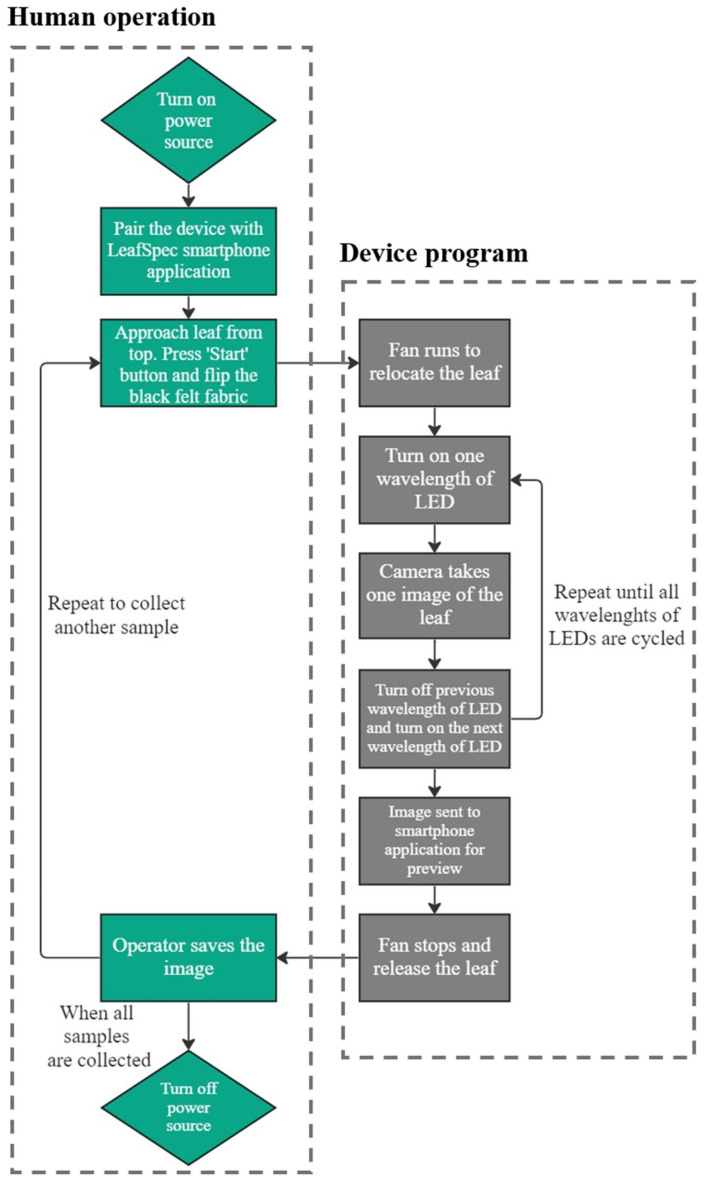
Operation Flowchart.

The flow chart in Figure 3 explains the operation flow of capturing images for one leaf. The first step of the imaging process is to turn on the power source and pair the device with LeafSpec smartphone App [28]. The smartphone App is used to manage the image captured from the device. Next, the operator finds the middle leaf of the top matured trifoliate of a soybean canopy and approaches the device to the leaf from directly above. 

Once the device is closer than 1 cm from the leaf, the operator presses the push button switch. Two DC fans will create airflow that attracts the leaf to the leaf barrier and hold the leaf against it. The operator then flips the black non-transparent felt fabric beneath the leaf to block the ambient light. The operator does not need to hold the fabric because the fabric is soft and light; the air flow created by the DC fans is also capable of holding it flat against the leaf barrier. With a one-second time delay after pressing the push button switch, the first wavelength of the LED turns on, and the camera captures an image. Such a process is repeated three times until all wavelengths of the LED are turned on once and a corresponding image is captured. Once completing the imaging sequence, two DC fans are turned off, and the leaf is released. Finally, the device transfers a preview image to the smartphone App via Bluetooth, and the operator examines the preview image to assure the entire leaf is captured.

The whole imaging process starts from pressing the toggle switch and ends as an image is transferred to the smartphone App, taking no more than five seconds. The time consumption for capturing one image has been drastically reduced five times compared to a current whole leaf imaging device, such as the imager developed by Wang [28]. Though the imaging speed has been increased, the data quality needs to be validated through sets of experiments.

## 3. Image Processing

With the image captured by the device, post-processing of the images is necessary before analyzing the nitrogen content. Since there was a gird of nylon thread used to hold the leaf specimen, it is necessary to reconstruct the leaf to recover the information to reach best image quality. Therefore, this section explains the image preprocessing procedure to improve the image quality, including calibration of uneven distribution of the light source, segmentation of the leaf from the backgrounds, the removal of nylon thread and recovery of those missing pixels.

### 3.1. Image Calibration

White referencing is used to compensate for the non-uniform distribution of the light source. Equation (1) is used to calibrate the raw image.

(1) $$Image_{cal}=\frac{Image_{raw}}{Image_{white}}\;$$

*Image_cal_* is the calibrated image; *Image_raw_* is the raw; and the *Image_white_* is the image of a white color board taken under each wavelength of light. Therefore, there are four *Image_white_* images used for corresponding *Image_raw_*. Same *Iamge_white_* was used for different samples as the nonuniformity is only related to the location of the LEDs, which was not changed among samples.

### 3.2. Image Segmentation

Color thresholding segmentation is used to segment the leaf from the background. As the background is pure black, the leaf area can be determined by calculating the *Greenness* of an image using the following Equation (2).

(2) $$Greenness=\frac{{\left(Image_{Green}\right)}^{2}}{Image_{Red}\;*\;Image_{Blue}}\;$$

After segmenting the green leaf from the background, a dilation operation followed by erosion operation was performed to smooth the edge of the leaf and to remove small objects from the image.

### 3.3. Removal of the Nylon Thread and Recovery of the Pixels

After removing the background from the images by color thresholding, the nylon threads were not removed. Because the values of the pixels corresponding to the nylon threads were always higher, due to the color and reflectivity, the thresholds used for segmenting black background were not high enough to segment the threads. It was possible to remove the threads simply by raising the thresholds, but the pixels corresponding to the leaf would be impacted as some leaf may have had intensities like the nylon threads.

**Figure 4:**
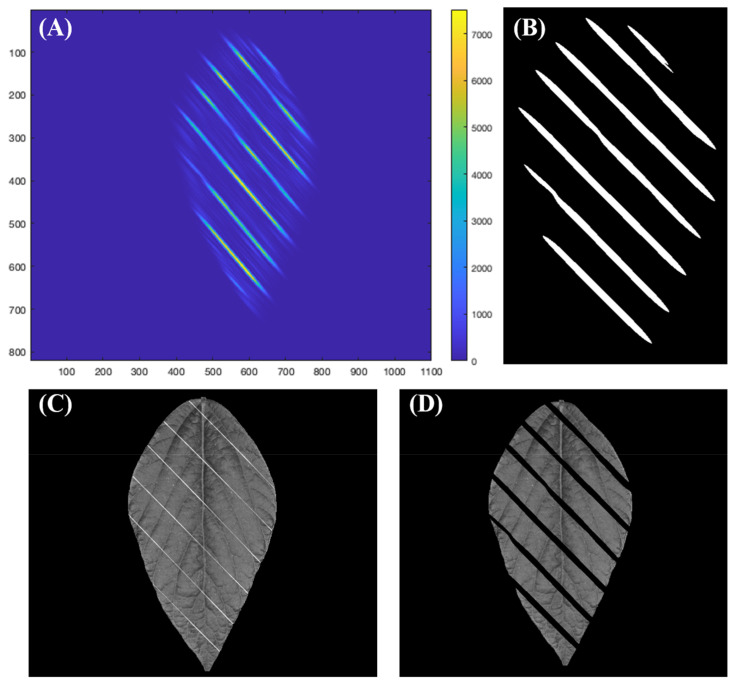
Segmentation procedure of nylon threads. (**A**) Magnitude map of Gabor filter recognizing aligned nylon threads. (**B**) Mask generated from the Gabor filter map. (**C**) Image before segmentation. (**D**) Image after segmentation.

Therefore, this study used a 2-D Gabor filter (Jain and Farrokhnia, 1991) integrated in MATLAB script (MathWorks, version 2021b) to segment the nylon threads with minimum impact to leaf pixels. The Gabor filter transforms the intensity of each pixel to the frequency response of an impulse signal. The frequency response contains two values: magnitude and phase. By varying the parameters of the impulse signal, objects with specific orientation will have different magnitude and phase; thus, the objects could be detected based on the phase and magnitude. In this study, the Gabor filter applies an impulse signal with wavelength parameter set as 5 and spatial aspect ratio parameter set as 0.1 was used to detect the nylon threads aligned at 45 degrees. Figure 4A shows the color map of the response magnitude obtained by applying the pre-described Gabor filter to the images captured by the device. Yellow color means the magnitude of the response is high, indicating that the orientation of the object in that pixel is more likely to be 45 degrees. On the other hand, a dark blue color means the magnitude of the response is low and the object in that pixel is likely to be orienting other ways. Therefore, the script used thresholding to filter the pixels with magnitude >1000 and obtained a mask, shown as Figure 4B. In Figure 4C the image before applying the mask is shown, and Figure 4D shows the image with nylon threads segmented.

After the pixel containing nylon threads is removed, the leaf becomes fragmentary and is not ideal for phenotyping. Therefore, the segmented image needs to be reconstructed to a whole leaf without missing pixels. Image inpainting has been a traditional technique to reconstruct images that are partially damaged or lost while a major part of the image is intact [29]. Popular image inpainting techniques are categorized into sequential-based, CNN-based and GAN-based, and the sequential-based techniques can be further categorized into diffusion-based and patch-based [30]. This study used the patch-based inpainting method to reconstruct the leaf because the techniques involving neural networks require a large data set to train and validate, which is out of the scope of this study. The inpainting technique is implemented by a function named *inpaintExemplar,* and it is included in the image-processing toolbox made by MATLAB (MathWorks, version 2021b). This function recognizes the gap area that needs to be inpainted and searches the remaining images for a best-matching patch to fill in the gap. By only using the pixels from the undamaged part of the image, the reconstructed area would have similar intensity with the rest of the image, which is crucial for multispectral imaging as many phenotypic parameters are calculated based on the image intensity under different lights. This function has two input arguments: the image to be inpainted and the mask of target regions. For this study, masks like Figure 4B are the masks of target regions, and the leaf images like Figure 4C are the images to be inpainted. There are two other parameters in the *inpaintExemplar* function: the Filling order parameter was set as tensor and the patch-size parameter was set as 9 × 9 pixels. The filling order parameter calculates the priority of each patch in the target region, and the patch size specifies the number of pixels in a patch. By reducing the patch size, the target region will be separated into more patches, resulting in more computing time. Based on our experiments, setting patch size as 9 is most cost-effective. 

**Figure 5:**
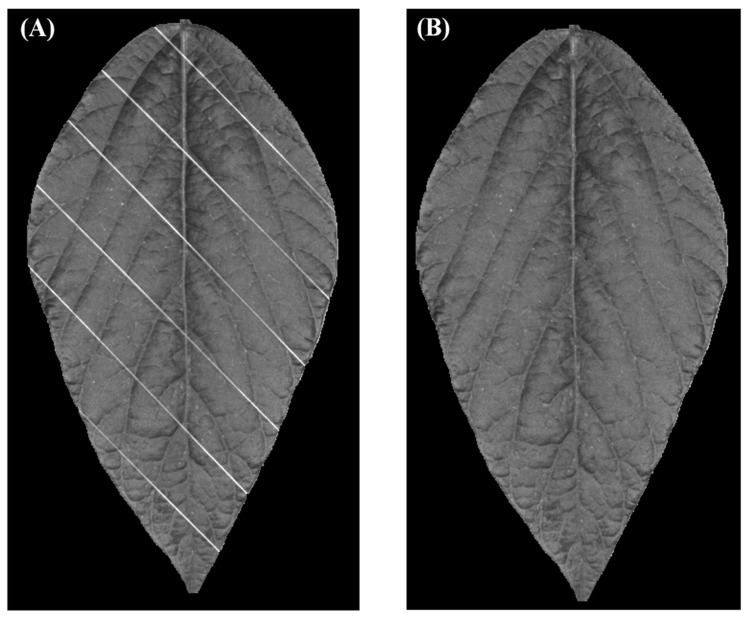
Image inpainting results. (**A**) Image before inpainting. (**B**) Image after inpainting.

Figure 5A,B shows an image before and after inpainting, respectively. In this figure, the pixels consisting of nylon threads are all replaced. Even though the inpainted pixels may not reflect the true information of that location on the leaf, it is still meaningful to inpaint those pixels because the nylon threads are purely noise, while the patch pixels from the leaf are relatively informative.

With the completion of the hardware assembly and software deployment, this study investigated the effectiveness of this device in separating nitrogen treatment, both under controlled environments and field conditions.

## 4. Validation Results of the Capability of Separating Nitrogen Treatment

Two rounds of imaging experiments were carried out to validate the capability of separating nitrogen treatments of this sensor. The first round imaged the plants grown in a controlled greenhouse environment. The second round imaged the plants grown in the field. 

### 4.1. Soybean Leaf Imaging in Controlled Environment

The first round of imaging was conducted using soybeans planted in plastic pots in the Lily greenhouse facility, located at Purdue University (West Lafayette, IN, USA). The soybean genotype was Asgrow 29XF1 from BAYER. The experiment used self-mixed growing medium. The medium was a mixture of BM2 seed germination and propagation mix made by Berger and pure sand, and the volume ratio was 2:1, respectively. Two nitrogen treatments with nitrogen parts per million (PPM) as 200 and 800 were applied to each genotype with 10 replicates for each treatment, which totaled to 20 plants. After about 60 days after seeding and the plants were at the V8 stage, the middle leaf of the top fully expanded trifoliate and the middle leaf of the lowest trifoliate was imaged by the newly developed device, resulting in 40 images.

Normalized Difference Vegetation Index (NDVI) has been proved to have strong correlation with the nitrogen content of a plant [31,32]. Since the device developed in this paper is multispectral and contains the wavelengths necessary to calculate the index, the *NDVI* across the leaf can be calculated by using the following Equation (3) [33].

(3) $$NDVI=\frac{Image_{NIR}-Image_{Red}}{Image_{NIR}+Image_{Red}}\;$$

**Figure 6:**
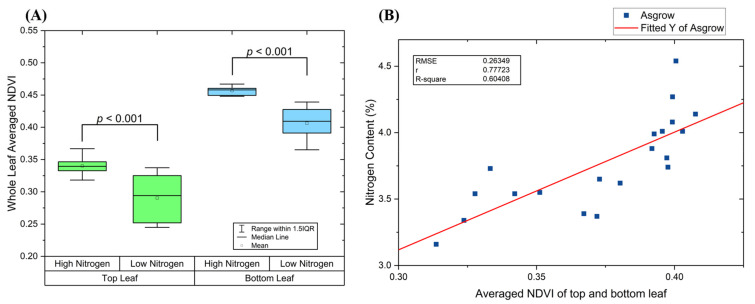
(**A**) Whole leaf averaged NDVI by leaf position and by nitrogen treatment. (**B**) Correlation of whole leaf averaged NDVI with the reference nitrogen content.

Then the whole leaf NDVI was averaged to obtain a single value. This value is used as an indicator to distinguish nitrogen treatment. The results are plotted in box chart shown as Figure 6A. The indicator obtained from top leaves has lower value than bottom leaves, which means younger leaves are relatively lighter green. Moreover, the range of the indicator for top leaves has larger range than bottom leaves. This is because top leaves are still developing, which results in more variance among different plants due to different growing stages. On the other hand, since the bottom leaves are fully developed and more stable across plants, the data ranges for two treatments are smaller.

A two-sample *t*-test between the same genotype was carried out to evaluate the device’s performance on detecting the effect of nitrogen treatment. Based on the indicator obtained from the bottom leaf, the *p*-value between high and low nitrogen treatment is 5 × 10^−6^ (*p* < 0.001). Moreover, the indicator obtained from the top trifoliate for Asgrow is 7 × 10^−4^ (*p* < 0.001). Therefore, based on the statistical results, it is reasonable to conclude that the whole leaf averaged NDVI captured from the device is effective on detecting the effect of nitrogen treatment.

After collecting the images for every subject, all the leaves from each plant were cut off and sent to laboratory (A&L Great Lakes Laboratories, Inc, Fort Wayne, IN, USA) for nitrogen content measurement. The laboratory results were used to determine the correlation between the indicator we obtained from the device and the nitrogen content of the plants. For each plant, we averaged the indicator collected from the leaf of top fully expanded trifoliate and the bottom leaf to characterize the whole plant. Figure 6B shows the fitted results for Asgrow. The reported R^2^ for the fitted model indicates a relatively strong correlation between the chemical reference data and the indicator measured by the newly developed device. 

This set of experiments have validated the device’s capability of detecting soybean nitrogen treatment with relatively high confidence. However, further experiments including more genotypes and treatment will be needed to validate the fitted model. The major source of error is the individual differences between each soybean canopy. As the soybeans are grown in the greenhouse, the microclimate will have effect on the growing of the canopy that could induce large variance to plant grow [25]. Another source of error is induced when inpainting the images as a small portion of the images are not the original leaf tissue. However, such noise is relatively small compared to current image-based spectroscopy phenotyping which is mostly completed under unstable lighting conditions and imaging angles.

### 4.2. Soybean Leaf Imaging in the Field

**Figure 7:**
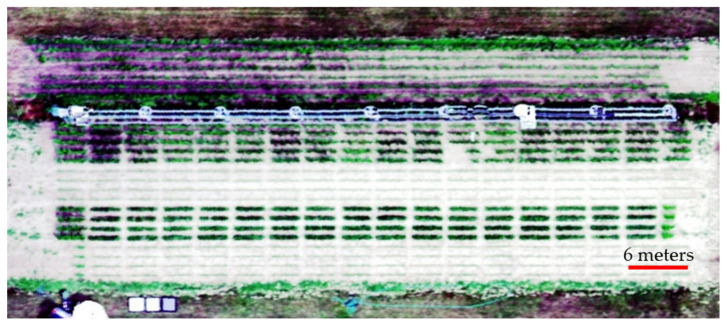
Field layout.

The second round of imaging took place at field 9C of Purdue Agronomy Center for Research and Education (ACRE) in West Lafayette, IN, USA in 2022. The field layout was shown in Figure 7. The experiment unit was a two-row plot, and each row was 3-m long. A randomized block design was conducted including two soybean varieties (P26T57E and P26T23E), two nutrient treatments (high nitrogen with 224 kg/ha and low nitrogen with 0 kg/ha), and four replicates per each soybean variety and nitrogen treatment. Nitrogen treatments were applied manually 21 days after planting when soybean plants were around the V3–V4 stage.

The imaging took place at V6–V7 stage, and only the middle leaf of the top fully expanded trifoliate was imaged using the device because the bottom leaves were not reachable due to the density of the soybean canopies. The imaging procedure was the same as imaging in the greenhouse. For each image, the NDVI heatmap was calculated and averaged to obtain a single indicator. Four randomly selected leaves were imaged from each plot. The results of these four images were averaged again to represent the plot to which they belonged.

**Figure 8:**
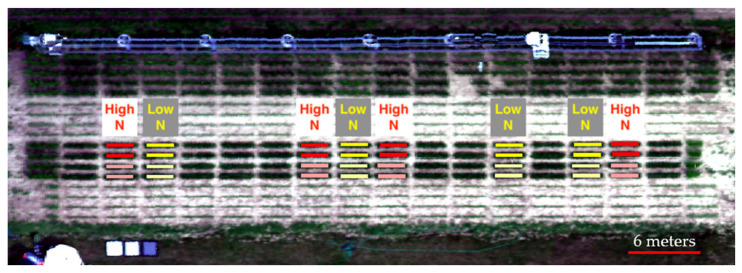
Reconstructed RGB image from hyperspectral data along with corresponding treatment.

Along with the individual leaf imaging, the field was imaged by an UAV (model: DJI M600) mounted with hyperspectral camera (model: Headwall Nano-Hyperspec VNIR (400–1000 nm)) to obtain remote sensing results of the field from 44 m above the field. The results are shown in Figure 8. This image is a reconstructed RGB image using the corresponding wavelength from the hyperspectral data; therefore, it does not reflect the true color of the field and is displayed as an example of the hyperspectral image.

A set of masks were created for each plot to calculate the average NDVI of that plot. Each rectangle mask includes 357 pixels and has identical shape, shown as red and yellow blocks in Figure 8. The location of each mask is slightly different to ensure it captures the center part of that plot and does not cover the edges of the plot.

**Figure 9:**
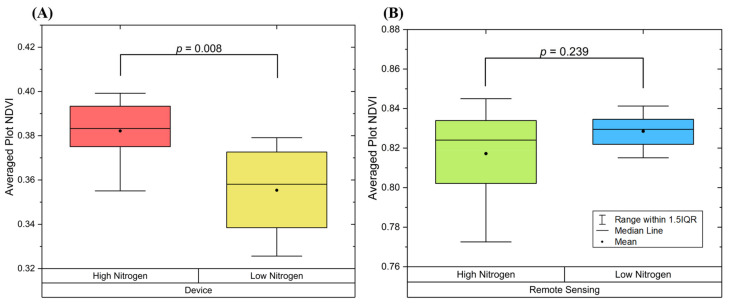
Results of distinguishing in-field nitrogen content. (**A**) Newly developed multispectral imaging device. (**B**) Hyperspectral imaging via UAV.

Figure 9 shows the results obtained from prescribed measurements along with a *p*-value calculated two-sample *t*-test. Based on the *p*-value, the images captured by the device separates the low and high nitrogen treatment, while images captured via remote sensing does not describe the treatment effectively. These results again prove the disadvantages of remote sensing, which includes too many noises from the environment, and the plant canopy could jeopardize the phenotyping results.

## 5. Discussions

In this study, the design of a new multispectral whole leaf imaging device is presented along with the experiment to validate the effectiveness of this new device. The purpose of developing this new device is to increase the sampling throughput without compromising the data quality. Compared to the previous developed device [18,27] the new design introduced active sensing method by using the airflow to grasp the leaf. Moreover, previous work focused on comparing the device with SPAD meter measurement. This correlation is not helpful enough in real-life scenario because users would like to know how to improve the condition (e.g., adding fertilizer, spray chemicals). Therefore, this study validated the device through differentiating nitrogen treatment, and this information could provide direct guidance on what must be done.

On the other hand, remote sensing still has higher priority compared to proximal sensing because remote sensing has the data acquisition speed that current proximal sensing technologies cannot meet. However, results from studies on farmland that have related NDVI of nitrogen content are still weak. Tilling et al. [34] reported a poor correlation with R^2^ = 0.19 in the study of correlating NDVI and nitrogen content. Saberioon and Gholizadeh [35] reported a 0.785 correlation coefficient between the remote sensing image and SPAD meter reading; however, the work did not link the data back to actual nitrogen content. In this study, a UAV was also used to image the field. However, based on the NDVI calculated from the remote sensing images, no significant difference was observed between different nitrogen treatments. Therefore, though remote sensing can cover larger areas at once, the information collected is not always useful for farmers or scholars to manage the field, and they may spend extra time to fully understand the field. 

Compared to remote sensing platforms, the newly developed proximal sensing device can effectively describe the nitrogen treatment in the field by only a few data points. Therefore, the total time consumption of using this device to image the field is not significantly increased. Furthermore, proximal sensing showed better capability in analyzing nutrient information, Song et al. [32] developed a new nitrogen index for soybean leaves based on color distribution on the leaf, which cannot be achieved by using remote sensing. Therefore, the newly developed device has very high potential to provide more valuable information as more studies will be conducted.

However, studying a huge field by human use of this device is still unachievable. In the future, with an automation system that can operate this device, the throughput would be further increased to match the throughput of remote sensing while having superior data quality.

## 6. Conclusions

In this study, a new type of multispectral proximal imaging device for soybean leaves has been developed. The device comprises a high-resolution camera, LEDs with 4 wavelengths and two DC fans. It is designed to block out ambient light while imaging and the LEDs inside the housing will provide consistent lighting conditions, which increases the signal-over-noise ratio and spatial resolution compared to remote sensing technologies. Moreover, this device used DC fans to create active airflow to reposition and flatten the leaves during imaging. This mechanism relieves the operator from laboring and time-consuming operations required by other proximal imaging devices and to improve image quality by removing noise caused by leaf angles. As a result, this device can image a soybean leaf in less than 5 s.

To evaluate the performance of this newly developed device, a greenhouse experiment, and an in-field experiment were carried out. In greenhouse experiments, the results show: (1) The device can clearly detect the nitrogen treatment with *p*-value < 0.01. (2) Data collected by the device shows a relatively strong linear correlation with laboratory measured nitrogen content. In field experiments, the remote sensing platform failed to distinguish the nitrogen treatment (*p* = 0.239) while the device successfully detects the effect of nitrogen treatment (*p* = 0.008). These experiments have proven the images captured by the device are informative for studying plant phenomics.

This new development has reduced the labor and time consumed in plant phenotyping without compromising the quality of the results. It has elevated the state-of-the-art of leaf-level multispectral imagers, and it could benefit wider populations. However, the data collection process both in field and controlled environment still requires human participation, which is labor and time-consuming. Therefore, it would be valuable to develop a robot system that can operate this newly developed sensor to further relieve human labor for even higher throughput and data quality.

## Data Availability

We would like to keep the data confidential.
